# Knowledge, Attitude and Conspiracy Beliefs of Healthcare Workers in Lebanon towards Monkeypox

**DOI:** 10.3390/tropicalmed8020081

**Published:** 2023-01-23

**Authors:** Diana Malaeb, Malik Sallam, Nesreen A. Salim, Mariam Dabbous, Samar Younes, Yves Nasrallah, Katia Iskandar, Matta Matta, Sahar Obeid, Souheil Hallit, Rabih Hallit

**Affiliations:** 1 College of Pharmacy, Gulf Medical University, Ajman P.O. Box 4184, United Arab Emirates; 2 School of Pharmacy, Lebanese International University, Beirut P.O. Box 146404, Lebanon; 3 Department of Pathology, Microbiology and Forensic Medicine, School of Medicine, The University of Jordan, Amman 11942, Jordan; 4 Department of Clinical Laboratories and Forensic Medicine, Jordan University Hospital, Amman 11942, Jordan; 5 Department of Translational Medicine, Faculty of Medicine, Lund University, 22184 Malmö, Sweden; 6 Prosthodontic Department, School of Dentistry, The University of Jordan, Amman 11942, Jordan; 7 Prosthodontic Department, Jordan University Hospital, Amman 11942, Jordan; 8 School of Education, Lebanese International University, Beirut P.O. Box 146404, Lebanon; 9 Department of Biomedical Sciences, School of Pharmacy, Lebanese International University, Bekaa P.O. Box 146404, Lebanon; 10 School of Medicine & Medical Sciences, Holy Spirit University of Kaslik, Beirut, Lebanon; 11 Pharmaceutical Sciences Department, School of Pharmacy, Lebanese International University, Bekaa P.O. Box 146404, Lebanon; 12 INSPECT-LB—National Institute of Public Health, Clinical Epidemiology and Toxicology-Lebanon, Beirut, Lebanon; 13 Department of Infectious and Tropical Diseases, Center Hospitalier de Melun, 77000 Melun, France; 14 Department of Social and Education Sciences, School of Arts and Sciences, Lebanese American University, Byblos, Lebanon; 15 Applied Science Research Center, Applied Science Private University, Amman 11931, Jordan; 16 Research Department, Psychiatric Hospital of the Cross, Jal Eddib P.O. Box 60096, Lebanon; 17 Department of Infectious Disease, Bellevue Medical Center, Mansourieh, Lebanon; 18 Department of Infectious Disease, Notre Dame des Secours, University Hospital Center, Byblos, Lebanon

**Keywords:** outbreak response, preparedness, KAP survey, healthcare, awareness, emergence of international concern, Middle East

## Abstract

The emergence of a monkeypox (MPOX) outbreak in 2022 represented the most recent recognizable public health emergency at a global level. Improving knowledge and attitude towards MPOX, particularly among healthcare workers (HCWs), can be a valuable approach in public health preventive efforts aiming to halt MPOX virus spread. The aim of the current study was to evaluate the knowledge and attitude of HCWs in Lebanon towards MPOX and to assess their conspiratorial attitude towards emerging virus infections (EVIs). The current study was based on a cross-sectional online survey distributed via Google Forms during September–December 2022 implementing a convenience sampling approach. The final study sample comprised a total of 646 HCWs: physicians (*n* = 171, 26.5%), pharmacists (*n* = 283, 43.8%), and nurses (*n* = 168, 26.0%), among others (*n* = 24, 3.7%). Variable defects in MPOX knowledge were detected, with a third of the participants having MPOX knowledge above the 75th percentile (*n* = 218, 33.7%). Satisfactory attitude towards MPOX (>75th percentile) was observed in less than a third of the participants (*n* = 198, 30.7%), while a quarter of the study sample endorsed conspiracy beliefs towards EVIs at a high level (>75th percentile, *n* = 164, 25.4%). Slightly more than two thirds of the participants agreed that MPOX vaccination should be used in disease prevention (*n* = 440, 68.1%). Better levels of MPOX knowledge and attitude were significantly associated with postgraduate education and older age. Physicians had significantly higher MPOX knowledge compared to other occupational categories. Less endorsement of conspiracies towards EVIs was significantly associated with male sex, occupation as a physician, and postgraduate education. Higher MPOX knowledge was associated with better attitude towards the disease. The current study showed unsatisfactory MPOX knowledge among Lebanese HCWs. Educational efforts can be valuable to improve the attitude towards the disease. Despite the relatively low level of embracing conspiracy beliefs regarding EVIs among HCWs in this study compared to previous studies, this area should be considered based on its potential impact on health-seeking behavior.

## 1. Introduction

In 2022, an unusual and unexpected rise in the number of human monkeypox cases was reported in over 100 nonendemic countries worldwide [1,2]. This zoonotic disease, known simply as monkeypox (MPOX), is caused by the monkeypox virus (MPXV), which was previously endemic in the tropical regions of Western and Central Africa [2,3]. Prior to the ongoing 2022 MPOX outbreak, limited spread of MPXV was reported primarily in endemic countries, with a secondary attack rate rarely exceeding 10% [3,4,5]. However, the dynamics of MPOX spread has drastically changed in 2022, with a cumulative number of confirmed cases exceeding 80,000 in more than 100 previously nonendemic countries [1,2,6]. This status obliged the World Health Organization (WHO) to declare the 2022 MPOX outbreak as a public health emergency of international concern on 23 July 2022 [7,8].

The MPXV was first documented in 1958 at an animal facility in Denmark and the first MPOX case in humans was reported in the Democratic Republic of the Congo in 1970 [2,9]. Monkeypox cases have largely been restricted to African countries. The disease progressed to countries outside the African territory in 2003. This was likely due to weakened immunity from cessation of the smallpox vaccination programs following variola eradication, greater human–wildlife interactions, and potential changes in MPXV that enabled sustained human-to-human transmission [10,11,12,13,14]. The changing epidemiology of MPOX mandates the provision of appropriate response measures [14]. These measures involve educational efforts aiming to increase awareness and knowledge of MPOX and offering vaccination for high-risk groups [2,15].

Although vaccines and treatments are available for MPOX management and prevention [16,17], the WHO highlighted the importance of effective public health control measures to halt the spread of MPXV [18]. This involves enhancing the capacity of healthcare workers (HCWs) to identify cases through implementing early diagnosis and providing systematic effective medical care [18,19]. Achievement of the required level of awareness about the clinical symptoms of MPOX is vital for early diagnosis and management, with subsequent prevention of forward MPXV transmission [20]. In this regard, the WHO, as well as national and local health agencies, have aimed to support and enhance the level of knowledge among HCWs [21,22,23]. Furthermore, prevention and treatment of infectious disease is a complicated process, as it often requires going beyond providing information, implementing preventive measures, which is dictated by the knowledge of the disease, attitudes toward prevention, and intentions to adopt recommended practices [21]. According to previous and recent studies involving health professionals, addressing gaps in MPOX knowledge can be considered an integral step to change attitudes and behavior [23,24,25,26,27,28]. When attitudes are grounded with high knowledge, they are associated with more enduring and better predictors of behaviors. Thus, the study of the baseline level of HCWs’ knowledge about an emerging infectious disease is of utmost value considering their high-risk status as frontline workers in disease response [15,29].

In light of the global emergency of MPOX prevalence, early prevention, timely detection, and quick response and management from HCWs in Lebanon will be extremely important. Although MPOX reported cases in Lebanon were only 24 as of 16 December 2022, it is crucial for HCWs to be knowledgeable and prepared for surveillance, diagnosis, and management of the disease. Lebanon is passing through severe economic crisis and its medical sector is struggling with shortages in medicine and medical equipment, which could raise the importance of applying the appropriate preventive measures [30,31]. In addition, the assessment of the relation between disease knowledge and attitude can have positive implications on the understanding of health-seeking behavior and adhering to preventive measures, especially in countries with a vulnerable healthcare system [32]. In turn, this can help to devise well-informed and tailored intervention measures necessary for the prevention of infectious disease spread [33,34]. Hence, we sought to assess the MPOX knowledge, attitude, as well as the conspiracy beliefs towards emerging virus infections among HCWs in Lebanon. Previous studies indicated the relevance of age, sex, and occupational category as relevant factors determining knowledge and attitude towards various infectious diseases among HCWs, as well as its association with varying prevalence of conspiracy beliefs in the context of COVID-19 [23,24,26,35,36].

## 2. Materials and Methods

### 2.1. Study Design and Setting

A cross-sectional online survey was conducted during September–December 2022 to assess knowledge, attitude, and practice of MPOX among HCWs in Lebanon. Invitations to complete an anonymous online survey were distributed by social media and instant messaging platforms and services (WhatsApp, Facebook, and Instagram).

Participants were informed that the study was anonymous and voluntary. Participants were then asked to provide their e-consent for study participation through a specific dichotomous question (i.e., Yes vs. No). Even though all individuals receiving the questionnaire and agreeing with the participation were able to complete the survey, only respondents who accurately compiled the questionnaire and fulfilled the main inclusion criteria (being a medical professional working as a medical doctor, pharmacist, or nurse) were included in subsequent analyses. All non-Lebanese HCWs and those who did not consent to participate were excluded from the study.

The study was approved by the ethics committee at the School of Pharmacy at the Lebanese International University, reference number: 2022RC-052-LIUSOP.

To ensure anonymity and confidentiality of the participants, personal data (e.g., name, address, e-mail address, or any personal information unnecessary to the survey) were neither requested, saved, nor tracked.

### 2.2. Sample Size Calculation

In this survey, 303 respondents were required as the minimum sample size based on a recent study which showed that around 27% of the physicians knew about MPOX and a confidence interval of 95% [23].

### 2.3. Study Instrument

The first section of the questionnaire consisted of demographic variables, while the remaining sections assessed MPOX knowledge, attitude, and conspiracy beliefs towards emerging virus infections. The demographic variables included age, sex, the highest educational level, residence area, and medical specialty (physician, pharmacist, nurse, and others (dentist and medical technician)). The knowledge, attitude, and conspiracy belief scales used in this study were based on measures developed for cross-sectional studies conducted among clinicians in Indonesia [25] and among students affiliated to health schools in Jordan [37].

#### 2.3.1. MPOX Knowledge Scale

The MPOX knowledge was assessed using a 17-item section as follows based on previous survey studies, with yes vs. no vs. I do not know as the possible responses [24,25,26]: “(1) MPOX is prevalent in the Middle East (incorrect); (2) MPOX is prevalent in Western and Central Africa (correct); (3) There is an outbreak of MPOX in the world (correct); (4) MPOX is caused by a virus (correct); (5) Human-to-human transmission of MPOX occurs through skin-to-skin contact (correct); (6) Human-to-human transmission of MPOX occurs through touching objects or surfaces that have been used by someone with MPOX (correct); (7) Human-to-human transmission of MPOX occurs through contact with respiratory secretions; (8) MPOX and smallpox have similar signs and symptoms (correct); (9) Skin rash is one of the signs or symptoms of MPOX (correct); (10) Pustule is one of the signs or symptoms of MPOX (correct); (11) Antibiotics are used to treat MPOX (incorrect), (12) Diarrhea is one of the signs or symptoms of MPOX (incorrect); (13) Vaccination is available to prevent MPOX (correct); (14) Young children less than 8 years of age are at increased risk for severe MPOX disease (correct); (15) Pregnant women are at increased risk for severe MPOX disease (correct); (16) Immunocompromised patients are at increased risk for severe MPOX disease (correct); and (17) Individuals with a history of atopic dermatitis or eczema are at increased risk for severe MPOX disease” [25,37]. Each correct response was given a score of 1, while incorrect responses and “I do not know” were scored as zero. The MPOX knowledge scale Cronbach’s alpha was 0.66.

#### 2.3.2. Emerging Virus Infections Conspiracy Scale

Regarding the assessment of the participants’ attitude towards conspiracy explanations of emerging virus infections, we adopted survey items from a study by Freeman et al. on coronavirus conspiracy beliefs, similar to the approach utilized by Sallam et al. in the context of MPOX [37,38]. The attitude towards conspiracy beliefs comprised a 12-item section, with 7-Likert scale possible responses (strongly disagree (1), disagree (2), somewhat disagree (3), neutral/no opinion (4), somewhat agree (5), agree (6), and strongly agree (7)). The following items comprised the Emerging Virus Infections Conspiracy Scale (EVICS) previously used in the context of MPOX knowledge [24,26,37,39]: (1) “I am skeptical about the official explanation regarding the cause of virus emergence”, (2) “I do not trust the information about the viruses from scientific experts”, (3) “Most viruses are man-made”, (4) “The spread of viruses is a deliberate attempt to reduce the size of the global population”, (5) “The spread of viruses is a deliberate attempt by governments to gain political control”, (6) “The spread of viruses is a deliberate attempt by global companies to take control”, (7) “Lockdowns in response to emerging infection are aimed for mass surveillance and to control every aspect of our lives”, (8) “Lockdowns in response to emerging infection are aimed for mass surveillance and to destabilize the economy for financial gain”, (9) “Lockdown is a way to terrify, isolate, and demoralize a society as a whole in order to reshape society to fit specific interests”, (10) “Viruses are biological weapons manufactured by the superpowers to take global control”, (11) “Coronavirus was a plot by globalists to destroy religion by banning gatherings”, and (12) “The mainstream media is deliberately feeding us misinformation about the virus and lockdown” [37,38].

Higher EVICS scores indicated a higher agreement level with conspiracy beliefs towards emerging virus infections [26,37]. The internal consistency of the scale was ensured by a Cronbach’s alpha value of 0.96.

#### 2.3.3. MPOX Attitude Scale

The MPOX attitude scale was a 9-item section, with 5-Likert scale possible responses (strongly disagree, disagree, neutral, agree, and strongly agree). The attitude items included: (1) In my opinion, early detection of MPOX virus can improve treatment and outcome; (2) In my opinion MPOX virus can be treated at home; (3) In my opinion, the MPOX virus transmission can be reduced by following the appropriate instructions provided; (4) In my opinion, if there is an available vaccine for MPOX, it should be used; (5) In my opinion, awareness of MPOX in society is sufficient; (6) In my opinion, MPOX can cause death; (7) In my opinion, MPOX virus can be transmitted from domestic pets to humans; (8) In my opinion, the authorities should restrict travel to and from the areas of outbreak of MPOX; and (9) In my opinion, authorities should isolate people infected with MPOX in private hospitals. The Cronbach’s alpha of the MPOX attitude scale was 0.57.

### 2.4. Statistical Analysis

The SPSS software (V22.0. Armonk, NY, USA: IBM Corp) was used for the statistical analysis. Cronbach’s alpha values were computed for internal reliability of the scales. The knowledge, conspiracy beliefs, and attitude scores were considered normally distributed since their skewness and kurtosis values were less than |1.96|. The Student *t* test was used to compare two means. The Pearson correlation test was used to correlate two continuous variables. Linear regressions were conducted, taking the knowledge, conspiracy beliefs, and attitude scores as dependent variables, respectively. Variables that showed a *p* < 0.250 in the bivariate analysis were entered as independent variables in the regression models. *p* < 0.050 was considered statistically significant.

## 3. Results

### 3.1. Sociodemographics and Other Characteristics of the Participants

A total of 646 HCWs completed the survey; their mean age was 32.64 ± 10.08 years and 53.3% were females; 43.8% of the participants were pharmacists and 62.4% lived in rural areas. When taking the 75th percentile as the cut-off point, the results showed that 218 (33.7%) had good MPOX knowledge, 164 (25.4%) had conspiracy beliefs, and 198 (30.7%) had a good attitude towards MPOX. Moreover, 45.5% of the sample were confident in diagnosing MPOX cases based on their current knowledge and skills, whereas 51.9% were confident in diagnosing MPOX cases based on the ability of their current facility to conduct diagnostic tests. At the end of the survey, 91.6% of the participants perceived the need to read more about MPOX. Other details regarding the sample are illustrated in Table 1.

### 3.2. The Overall MPOX Knowledge, Conspiracy Beliefs, and Attitude towards MPOX in the Study Sample

The description of each item’s response of the knowledge, attitude, and conspiracy beliefs scales can be found in Table 2, Table 3 and Table 4, respectively.

### 3.3. Bivariate Analysis

The results of the bivariate analysis are summarized in Table 5 and Table 6.

Having a postgraduate education was significantly associated with higher MPOX knowledge and better attitude towards the disease. In addition, postgraduate education was associated with lower levels of conspiracy beliefs towards emerging virus infections, while female sex was associated with higher levels of embracing these conspiracy beliefs. Physicians had the highest mean scores of MPOX knowledge and attitude and the lowest mean conspiracy belief scores compared to other HCWs. Finally, higher MPOX knowledge was significantly associated with better attitude, whereas older age was significantly associated with better MPOX knowledge and attitude.

### 3.4. Multivariate Analysis

Physicians (β = 2.92), pharmacists (β = 1.74), and nurses (β = 2.39) had more knowledge about MPOX compared to dentists and technicians (Table 7, Model 1).

Having a postgraduate education (β = −3.88) compared to having a bachelor degree, and physicians (β = −20.49) and nurses (β = −13.83) compared to dentists and technicians were significantly associated with less conspiracy beliefs, whereas older age (β = 0.20) was significantly associated with higher endorsement of conspiracy beliefs (Table 7, Model 2).

Physicians (β = 3.79) and nurses (β = 3.34) compared to dentists and technicians, and higher MPOX knowledge (β = 0.38) and lower level of conspiracy beliefs (β = 0.02) were significantly associated with better MPOX attitude (Table 7, Model 3).

## 4. Discussion

The major findings of this study, which was conducted amid the ongoing 2022 MPOX multi-country outbreak, can be summarized as follows: first, significant gaps in knowledge about MPOX were revealed in this study, with merely more than a third of the participants having satisfactory MPOX knowledge. Second, about a quarter of the study sample harbored specific conspiracy beliefs regarding emerging virus infections and their subsequent measures. Third, a majority of the study respondents displayed good attitude towards MPOX. Fourth, satisfactory MPOX knowledge was significantly correlated with good attitude towards the disease. Finally, multivariate analyses showed that occupational category was an important determinant of MPOX knowledge, attitude, and conspiratorial attitude towards emerging virus infections.

Concerning the gaps in MPOX knowledge among the Lebanese HCWs included in this study, several issues were revealed. These defects in MPOX knowledge should be considered in the efforts aiming to increase HCWs preparedness for outbreak response in the country despite the limited number of cases (24 confirmed cases) that has been recorded in Lebanon as of 22 December 2022 [6]. For example, MPOX vaccination can have an important role in the preventive efforts for high-risk groups, including HCWs [19]. Additionally, satisfactory knowledge and positive attitude towards MPOX vaccination can affect HCWs’ recommendation of the vaccine for other high-risk groups (e.g., men who have sex with men (MSM)) [28,40]. Specifically, and in the context of MPOX vaccination intention among the general public in the U.S., Winters et al. reported that the most trusted sources of information regarding the outbreak were HCWs [41].

In this study, only 50% of the respondents were aware that vaccination is available to prevent MPOX. Considering the survey timing (November–December 2022), this result shows that gaps in MPOX knowledge still exist despite the intensive awareness campaigns and the growing literature on MPOX prevention [2,10,42]. Previous studies showed variable levels of MPOX knowledge and attitude towards MPOX vaccination in different countries worldwide [23,26,37,39,43,44,45,46]. For example, a survey study among Italian physicians, which was conducted in May 2022, showed a generally favorable attitude towards MPOX vaccination [23]. Another survey study among Saudi physicians that concluded on May 2022 showed a much higher rate of awareness of MPOX vaccine availability (70%), compared to the results of the current study (50%) [27]. Nevertheless, the findings of the current study concerning the knowledge of MPOX vaccine availability was higher compared to results of the study among HCWs in Jordan (<33%) [26].

The issue of awareness of vaccine availability and subsequent willingness to get vaccinated is of prime importance in the post-COVID-19 era [47]. The phenomenon of vaccination hesitancy flared up during COVID-19, even among health professionals [48,49,50]. In the context of the ongoing MPOX outbreak, the latest WHO recommendations for primary vaccination listed HCWs at risk of repeated MPXV exposure (together with MSM, sex workers, and other individuals with high-risk sexual behavior) [51]. Nevertheless, negative attitude towards MPOX vaccination has been reported in recent studies. For example, a survey study among HCWs in Czech Republic that was conducted in September 2022 showed that only 9% of the study sample agreed to receive MPOX vaccination [43]. Another recent study among Nigerian HCWs showed that only 31% of the study sample had confidence in MPOX vaccination [45]. The aforementioned study by Ghazy et al. also showed complacency towards the disease can be an important factor in reluctance to receive MPOX vaccination [45]. In the current study, only 28% of the participants agreed that MPOX can cause death among the infected individuals, highlighting the widespread view of MPOX as a remotely dangerous disease. Additionally, the fraction of the respondents in this study who agreed that MPOX vaccination should be utilized was 68%, suggesting that MPOX vaccination acceptability and possible recommendation is not universally embraced by Lebanese HCWs, at least in the study sample. On the other hand, a recent study among Chinese HCWs showed that only 10% of the participants were unwilling to receive MPOX vaccination [44], suggesting the importance of culture and context as determinants of vaccine willingness.

Other areas where discernible gaps in HCWs’ knowledge were found in this study included the recognition of lack of direct antibiotic utility in the treatment of MPOX, which is a virus infection. Such a result was also found in recent studies among HCWs in Jordan and Kuwait, in addition to students in health schools in Jordan [24,26,37]. This issue is of particular interest in the Middle East countries where self-medication practices are commonplace in addition to the high prevalence of antimicrobial drug resistance [52,53,54,55,56,57].

In this study, an overall similar level of MPOX knowledge was noticed among the study group compared to the results of recent studies conducted among HCWs in Jordan, Kuwait, and Saudi Arabia [24,26,27]. Additionally, the level of MPOX knowledge was higher compared to the results reported among the general populations in the Middle East region, highlighting the role that HCWs can play in community education, which is needed to contain the spread of MPXV [15,58,59].

Gaps in knowledge detected in the current study alongside other recent studies in the region highlight the importance of timely information delivery that can be critical in the efforts aiming to control the global MPOX outbreak. Noticeable knowledge defects in various aspects of the disease, including treatment and vaccination, were reported among physicians in Saudi Arabia by Alshahrani et al. [27]. In a recent study among HCWs in Jordan, less than 70% correct responses were found for a majority of MPOX knowledge items used in the study [26]. A similar result was also reported among HCWs in Kuwait, with a higher level of knowledge among physicians compared to other HCW occupational categories. In this study, physicians displayed a significantly higher level of knowledge as well; nevertheless, knowledge gaps were still noticeable among physicians in various studies and this pattern was reported in this study. Consistent with our results, only 33% of physicians in a recent study among Turkish physicians were reported to have good MPOX knowledge [60]. Addressing these knowledge gaps can be valuable, since HCWs have a key role in community education and preparedness for outbreak response, particularly among high-risk groups (e.g., MSM and people living with HIV infections/AIDS (PLWHA)) [40]. This includes educational efforts necessary to recognize unusual presentation of the disease, including its genital manifestations [61].

The proposed reasons behind low level of MPOX knowledge reported in different recent studies include the lack of inclusion of educational material in medical curricula tackling emerging and often neglected tropical infections in various countries worldwide [23,25]. In addition, the relatively low number of MPOX cases in the Middle East and its concentration among certain risk groups (e.g., MSM, which is considered a hidden and stigmatized sub-population in the region) may have resulted in less interest to acquire knowledge regarding this particular subject [6,62,63].

Boosting the level of knowledge regarding an emerging infection can have a positive effect on confidence in diagnosis and management. The significant association between confidence in diagnosis and management in relation to MPOX knowledge has been reported in past and recent studies in the countries of the Middle East, as well as among Indonesian physicians [24,26,64]. Additionally, and based on the finding in this study, positive attitude towards MPOX was significantly correlated with better knowledge, highlighting the need to focus on improving MPOX knowledge among HCWs. This issue is of particular importance in Lebanon, considering the ongoing economic difficulties that compromised the delivery of optimum healthcare services [30].

The assessment of the prevalence of specific conspiracy beliefs among HCWs in Lebanon was an additional objective of this study. Such an aim was relevant considering the accumulating evidence of noticeable circulation of rumors and misinformation when a novel infectious disease emerges [65]. This observation was evident in the context of MPOX in the Middle East, as reflected particularly in the social media platforms [66]. Thus, in the context of MPOX emergence, evaluating the content and prevalence of such specific conspiracies can be an important step in the efforts needed to fight the spread of misinformation considering the previous evidence of its possible harmful health and psychologic impact [67,68,69].

In this study, the prevalence of conspiracy ideas towards virus emergence was lower compared to similar studies among HCWs in Jordan and Kuwait with the utilization of the same survey assessment tool (about 20% vs. more than 50% in the previous studies) [24,26]. Additionally, the results of this study showed much lower prevalence of EVI conspiracies compared to the results obtained among the general public in Lebanon by Youssef et al., which showed a general prevalence of specific conspiracies at a rate of 59% [70]. Possible explanation of this result can be related to survey timing, since the Jordanian and Kuwaiti studies, as well as the Lebanese study among the general public, were conducted early on during the course of the 2022 MPOX outbreak. During the early phase of MPOX outbreak, and reminiscent of the early phase of COVID-19 pandemic, conspiracy theories emerged surrounding the origin and spread of MPXV [71]. A recent study that investigated the Twitter content amid the 2022 MPOX multi-country outbreak found that negative sentiment tweets were more common compared to positive sentiment tweets despite the dominance of neutral ones [42].

A recurring pattern that was reported in this study is the variable prevalence of endorsing various conspiracy ideas regarding virus emergence, with the highest prevalence reported for the following items: “viruses are biological weapons manufactured by the superpowers to take global control” and “lockdowns in response to emerging infections are aimed for mass surveillance and to control every aspect of our lives”, with an agreement level of 25% for both items. Consistent with this result, the idea that viruses are biological bioweapons was also highly prevalent in the studies that used the same survey instrument, regardless of the tested population (HCWs, general public, and university students) [24,26,37,39]. Therefore, dismantling such a wrong belief should be conducted, with a focus on educational efforts highlighting the natural origin of infectious diseases via cross-species transmission from animal sources [72].

Regarding the attitude towards MPOX, a generally positive attitude was found among the study respondents. A majority of participants (72%) agreed that early detection of MPXV can improve treatment and outcome, and also with the idea that MPXV transmission can be reduced through following the appropriate instructions provided (74%). Importantly, a more positive attitude was observed among physicians compared to other HCW categories included in the study. Furthermore, in this study, positive attitude was significantly correlated with better knowledge and lower embrace of conspiracy beliefs. This result highlights the importance of previous evidence suggesting that tackling knowledge would be an essential step in changing attitudes towards disease and should be considered in the interventional measures aiming to control MPOX outbreak [73,74].

For both MPOX knowledge and attitude, postgraduate education and occupation as a physician were associated with higher knowledge and a more positive attitude towards the disease. This result is understandable considering the importance of education (and possibly seniority level) in outbreak response and community education. This pattern was also reported at a smaller level among Malaysian dental students, where clinical students were reported to have better knowledge [75]. This result highlights the importance of tailoring the educational campaigns among other intervention measures to the needs of each occupational category and based on the educational level for each category. This finding was in line with what has been reported in the Jordanian and Kuwaiti studies, suggesting the generalizability of such a pattern [24,26].

In multivariate analysis, the results confirmed the importance of occupational category as a determinant of MPOX knowledge, embrace of EVI conspiracies, and attitude towards MPOX. Additionally, the educational level was an independent determinant of EVI conspiracies. Furthermore, the attitude towards MPOX was associated with MPOX knowledge and embrace of conspiracies, highlighting the importance of addressing both issues to reach a favorable attitude towards the disease, as previously discussed.

### Study Strengths and Limitations

The current study represents the first study to analyze the knowledge and attitude of HCWs in Lebanon towards MPOX. The results of this study could have important implications in terms of developing and implementing policies and intervention measures necessary for the prevention and management of the disease. Additionally, the assessment of the prevalence and scope of conspiracies towards emerging virus infections was an additional strength of this study, taking into account the previous evidence of its harmful impact on health behavior [35,69,76]. The study findings showed substantial knowledge gaps and erratic perceptions of MPOX risks, underscoring the need for MPOX awareness campaigns for first-line Lebanese HCWs. Hence, educational interventions with clinicians should address inadequate knowledge to support correct diagnosis and treatment. Additionally, training and more in-depth continuous education about this topic and any suspected emergent diseases are crucial.

The limitations of the study included (1) the sampling approach, which depended on convenience sampling, with subsequent risk of selection bias; (2) selection bias was also evident by the participation of a majority of pharmacists, which should be considered in the attempt to generalize the results of the study. Thus, analysis per occupational category can be a better approach to draw valid conclusions regarding the study aims; (3) an inherent limitation of the cross-sectional studies was inevitable in the current study, which can be viewed as a snapshot description of knowledge and attitude that can change over time; (4) the self-reported nature of responses should be taken into account as well, with a possibility of social desirability bias; and, finally, (5) the knowledge and attitude scales had low Cronbach’s alpha values, probably because they are non-validated tools.

## 5. Conclusions

The opportunity to limit the spread of MPOX should not be missed. The vigilant surveillance strongly relies on the role of HCWs. Educational campaigns to strengthen HCWs’ knowledge of MPOX and to raise awareness of disease can be valuable to boost their confidence in diagnosis and management of the disease. This is of particular importance in countries with a fragile healthcare system, including Lebanon. The finding that better MPOX knowledge was significantly correlated with positive attitude to MPOX and lower conspiracies towards emerging infections highlights the need for HCWs’ proper education on this subject. Considering the urgency of the current outbreak, intensified educational campaigns are necessary, with a special focus on raising awareness regarding MPOX vaccine utility and lack of antibiotic usefulness to treat the disease. Despite the relatively low prevalence of conspiratorial beliefs in this study, more than one fifth of the participants agreed with such beliefs. This is a worrisome result considering the previous evidence of the negative impact of such a phenomenon on health-seeking behavior.

## Figures and Tables

**Table 1:** Sociodemographic and other characteristics of the participants (*n* = 646).

Characteristic	Number (%)
**Sex**	
Male	302 (46.7%)
Female	344 (53.3%)
**Educational level**	
Bachelor	285 (44.1%)
Postgraduate	361 (55.9%)
**Residence**	
Urban	243 (37.6%)
Rural	403 (62.4%)
**Occupation**	
Physician	171 (26.5%)
Pharmacist	283 (43.8%)
Nurse	168 (26.0%)
Other (dentist, medical technician)	24 (3.7%)
**Scale variables**	**Mean** ** ± SD ^2^**
Age, years	32.64 ± 10.08
Monkeypox Knowledge	12.50 ± 3.48
Conspiracy beliefs ^1^	37.29 ± 16.69
Attitude towards monkeypox	31.50 ± 4.93

^1^ Assessed using Emerging Virus Infections Conspiracy Scale (EVICS) [37]; ^2^ SD: standard deviation.

**Table 2:** Description of the knowledge scale items’ responses.

Monkeypox Knowledge Items	No	I Do Not Know	Yes
MPOX is prevalent in the Middle East	**268 (41.5%)**	110 (17.0%)	268 (41.5%)
MPOX is prevalent in Western and Central Africa	58 (9.0%)	88 (13.6%)	**500 (77.4%)**
There is an outbreak of human MPOX in the world	137 (21.2%)	89 (13.8%)	**420 (65.0%)**
MPOX is caused by a virus	66 (10.2%)	56 (8.7%)	**524 (81.1%)**
Human-to-human transmission of MPOX occurs through skin-to-skin contact	49 (7.6%)	41 (6.3%)	**556 (86.1%)**
Human-to-human transmission of MPOX occurs through touching objects or surfaces that have been used by someone with MPOX	109 (16.9%)	60 (9.3%)	**477 (73.8%)**
Human-to-human transmission of MPOX occurs through contact with respiratory secretions	112 (17.3%)	200 (31.0%)	**334 (51.7%)**
MPOX and smallpox have similar signs and symptoms	123 (19.0%)	107 (16.6%)	**416 (64.4%)**
Skin rash is one of the signs or symptoms of human MPOX	52 (8.0%)	52 (8.0%)	**542 (83.9%)**
Pustule is one of the signs or symptoms of human MPOX	115 (17.8%)	70 (10.8%)	**461 (71.4%)**
Antibiotics are used to treat human MPOX	**168 (26.0%)**	118 (18.3%)	360 (55.7%)
Diarrhea is one of the signs or symptoms of human MPOX	**222 (24.4%)**	158 (24.5%)	266 (41.2%)
Vaccination is available to prevent human MPOX	206 (31.9%)	118 (18.3%)	**322 (49.8%)**
Who is at increased risk for severe MPOX disease?			
Young children less than 8 years of age are at increased risk for severe MPOX disease	152 (23.5%)	54 (8.4%)	**440 (68.1%)**
Pregnant women are at increased risk for severe MPOX disease	170 (26.3%)	74 (11.5%)	**402 (62.2%)**
Immunocompromised patients are at increased risk for severe MPOX disease	94 (14.6%)	37 (5.7%)	**515 (79.7%)**
Individuals with a history of atopic dermatitis or eczema are at increased risk for severe MPOX disease	196 (30.3%)	86 (13.3%)	**364 (56.3%)**

Numbers in bold indicate correct responses; MPOX: monkeypox.

**Table 3:** Description of the emerging virus conspiracy beliefs scale items’ responses.

Emerging Virus Conspiracy Beliefs Scale Items	Strongly Disagree	Disagree	Somewhat Disagree	Neutral	Somewhat Agree	Agree	Strongly Agree
I am skeptical about the official explanation regarding the cause of virus emergence	152 (23.5%)	176 (27.2%)	56 (8.7%)	129 (20.0%)	68 (10.5%)	52 (8.0%)	13 (2.0%)
I do not trust the information about the viruses from scientific experts	153 (23.7%)	226 (35.0%)	89 (13.8%)	85 (13.2%)	48 (7.4%)	30 (4.6%)	15 (2.3%)
Most viruses are man-made	128 (19.8%)	203 (31.4%)	93 (14.4%)	103 (15.9%)	55 (8.5%)	47 (7.3%)	17 (2.6%)
The spread of viruses is a deliberate attempt to reduce the size of the global population	127 (19.7%)	195 (30.2%)	73 (11.3%)	111 (17.2%)	72 (11.1%)	50 (7.7%)	18 (2.8%)
The spread of viruses is a deliberate attempt by governments to gain political control	115 (17.8%)	178 (27.6%)	91 (14.1%)	124 (19.2%)	70 (10.8%)	48 (7.4%)	20 (3.1%)
The spread of viruses is a deliberate attempt by global companies to take control	118 (18.3%)	175 (27.1%)	95 (14.7%)	110 (17.0%)	78 (12.1%)	43 (6.7%)	27 (4.2%)
Lockdowns in response to emerging infections are aimed for mass surveillance and to control every aspect of our lives	118 (18.3%)	159 (24.6%)	92 (14.2%)	119 (18.4%)	74 (11.5%)	53 (8.2%)	31 (4.8%)
Lockdowns in response to emerging infections are aimed for mass surveillance and to destabilize the economy for financial gain	106 (16.4%)	171 (26.5%)	93 (14.4%)	131 (20.3%)	71 (11.0%)	52 (8.0%)	22 (3.4%)
Lockdown is a way to terrify, isolate, and demoralize a society as a whole in order to reshape society to fit specific interests	110 (17.0%)	182 (28.2%)	94 (14.6%)	122 (18.9%)	71 (11.0%)	40 (6.2%)	27 (4.2%)
Viruses are biological weapons manufactured by the superpowers to take global control	100 (15.5%)	167 (25.9%)	100 (15.5%)	120 (18.6%)	78 (12.1%)	50 (7.7%)	31 (4.8%)
Coronavirus was a plot by globalists to destroy religion by banning gatherings	115 (17.8%)	156 (24.1%)	103 (15.9%)	133 (20.6%)	55 (8.5%)	48 (7.4%)	36 (5.6%)
The mainstream media is deliberately feeding us misinformation about the virus	108 (16.7%)	140 (21.7%)	119 (18.4%)	143 (22.1%)	61 (9.4%)	39 (6.0%)	36 (5.6%)

**Table 4:** Description of the monkeypox attitude scale items’ responses.

Monkeypox Attitude Scale Items	Strongly Disagree	Disagree	Neutral	Agree	Strongly Agree
In my opinion, early detection of MPOX virus can improve treatment and outcome	30 (4.6%)	44 (6.8%)	110 (17.0%)	209 (32.4%)	253 (39.2%)
In my opinion, MPOX virus can be treated at home	26 (4.0%)	87 (13.5%)	162 (25.1%)	261 (40.4%)	110 (17.0%)
In my opinion, the MPOX virus transmission can be reduced by following the appropriate instructions provided	21 (3.3%)	44 (6.8%)	104 (16.1%)	264 (40.9%)	213 (33.0%)
In my opinion, if there is an available MPOX vaccine for the disease, it should be used	19 (2.9%)	59 (9.1%)	128 (19.8%)	236 (36.5%)	204 (31.6%)
In my opinion, awareness of MPOX disease in society is sufficient	129 (20.0%)	165 (25.5%)	142 (22.0%)	115 (17.8%)	95 (14.7%)
In my opinion, MPOX can cause death	107 (16.6%)	170 (26.3%)	191 (29.6%)	101 (15.6%)	77 (11.9%)
In my opinion, MPOX virus can be transmitted from domestic pets to humans	69 (10.7%)	107 (16.6%)	179 (27.7%)	155 (24.0%)	136 (21.1%)
In my opinion, the authorities should restrict travel to and from the areas of outbreak of MPOX disease	36 (5.6%)	55 (8.5%)	164 (25.4%)	224 (34.7%)	167 (25.9%)
In my opinion, authorities should isolate people infected with MPOX in private hospitals	36 (5.6%)	57 (8.8%)	165 (25.5%)	213 (33.0%)	175 (27.1%)

MPOX: monkeypox.

**Table 5:** Bivariate analysis of factors associated with the knowledge, conspiracy beliefs, and attitude scores.

	Knowledge	*p*	Conspiracy Beliefs	*p*	Attitude	*p*
Sex		0.786		**0.003**		0.167
Male	11.13 ± 2.96		35.21 ± 16.88		31.78 ± 5.05	
Female	11.06 ± 3.10		39.12 ± 16.33		31.24 ± 4.83	
Education		**0.010**		**<0.001**		**<0.001**
Bachelor	10.75 ± 3.23		41.62 ± 15.87		30.48 ± 5.08	
Postgraduate	11.37 ± 2.85		33.87 ± 16.55		32.30 ± 4.67	
Residence		0.379		0.617		0.266
Urban	10.96 ± 2.92		37.72 ± 17.93		31.77 ± 4.89	
Rural	11.18 ± 3.10		37.02 ± 15.91		31.33 ± 4.96	
Occupation		**<0.001**		**<0.001**		**<0.001**
Physician	11.95 ± 2.34		28.47 ± 14.25		33.56 ± 4.04	
Pharmacist	10.64 ± 3.48		43.38 ± 15.05		29.68 ± 4.66	
Nurse	11.32 ± 2.32		34.52 ± 16.80		32.80 ± 4.83	
Other	8.88 ± 4.10		47.67 ± 16.66		29.00 ± 5.95	

Numbers in bold indicate significant *p* values.

**Table 6:** Correlation of continuous variables between each other.

Variable	1	2	3	4
1. MPOX Knowledge ^1^	-	-	-	-
2. EVI Conspiracy beliefs ^2^	−0.01	-	-	-
3. Attitude	0.36 ***	−0.06	-	-
4. Age	0.11 **	−0.08	0.13 **	-

** *p* <0.010; *** *p* <0.001; ^1^ MPOX: monkeypox; ^2^ EVI: emerging virus infection.

**Table 7:** Multivariable analyses.

Model 1: Linear Regression Taking the Knowledge Score as the Dependent Variable (ENTER Method) (R^2^ = 0.055)				
	Beta	β	*p*	95% CI
Education (postgraduate vs. bachelor *)	0.14	0.02	0.595	−0.38; 0.66
Physician vs. other *	2.92	0.43	**<0.001**	1.62; 4.23
Pharmacist vs. other *	1.78	0.29	**0.005**	0.54; 3.02
Nurse vs. other *	2.44	0.35	**<0.001**	1.16; 3.71
Age	0.01	0.03	0.499	−0.02; 0.04
**Model 2: Linear regression taking the conspiracy beliefs score as the dependent variable (ENTER method) (R^2^ = 0.177)**				
Sex (females vs. males *)	1.81	0.05	0.154	−0.68; 4.29
Education (postgraduate vs. bachelor *)	−3.88	−0.12	**0.005**	−6.56; −1.19
Physician vs. other *	−20.49	−0.54	**<0.001**	−27.25; −13.73
Pharmacist vs. other *	−5.60	−0.17	0.086	−12.00; 0.80
Nurse vs. other *	−13.83	−0.36	**<0.001**	−20.41; −7.24
Age	0.20	0.12	**0.005**	0.06; 0.33
Knowledge	0.31	0.06	0.085	−0.04; 0.65
**Model 3: Linear regression taking the attitude score as the dependent variable (ENTER method) (R^2^ = 0.212)**				
Sex (females vs. males *)	0.02	0.002	0.958	−0.70; 0.74
Education (postgraduate vs. bachelor *)	0.54	0.05	0.177	−0.24; 1.32
Physician vs. other *	3.79	0.34	**<0.001**	1.78; 5.80
Pharmacist vs. other *	0.30	0.03	0.753	−1.56; 2.16
Nurse vs. other *	3.34	0.30	**0.001**	1.41; 5.27
Age	−0.01	−0.02	0.566	−0.05; 0.03
Knowledge	0.38	0.27	**<0.001**	0.28; 0.48
Conspiracy beliefs	0.02	0.08	**0.047**	0.001; 0.05

* Reference group; Beta = unstandardized beta; β = standardized beta; CI: confidence interval; numbers in bold indicate significant *p* values.

## Data Availability

The data presented in this study are available on request from the corresponding author (S.H.).
